# PEDOT:PSS—A Key Material for Bioelectronics

**DOI:** 10.1002/advs.202513480

**Published:** 2025-12-23

**Authors:** Alan Eduardo Ávila Ramírez, David Pieter van der Laan, Mustafeez Bashir Shah, Liwen Wang, Erica Zeglio, Achilleas Savva

**Affiliations:** ^1^ Division of Nanobiotechnology Department of Protein Science Science for Life Laboratory School of Engineering Sciences in Chemistry Biotechnology and Health KTH Royal Institute of Technology Solna 171 65 Sweden; ^2^ Department of Microelectronics Faculty of Electrical Engineering Mathematics and Computer Science Delt University of Technology Mekelweg 4 CD Delft 2628 the Netherlands; ^3^ Wallenberg Initiative Materials Science for Sustainability Department of Chemistry Stockholm University Stockholm 114 18 Sweden; ^4^ AIMES – Center for the Advancement of Integrated Medical and Engineering Sciences at Karolinska Institutet and KTH Royal Institute of Technology Stockholm Sweden

**Keywords:** bioelectronics, conducting polymers, organic electronics, organic mixed‐ionic electronic conductors, PEDOT:PSS

## Abstract

Bioelectronics is a rapidly evolving interdisciplinary field that integrates principles of electrical engineering, materials science, and biology to develop electronic interfaces capable of recording and stimulating biological activity of the human body. The conducting polymer poly(3,4‐ethylenedioxythiophene):poly(styrenesulfonate) (PEDOT:PSS) has emerged as a key bioelectronic material due to its unique properties, processing versatility, and biocompatibility. This work provides an overview of PEDOT:PSS‐based bioelectronic interfaces and their growing potential in clinical applications. The historical development of PEDOT:PSS is first traced, highlighting its rise as one of the most successful materials in organic bioelectronics. The fundamental properties that make PEDOT:PSS particularly well‐suited for bioelectronic interfaces are then examined, with a focus on how these properties can be precisely tuned through advanced processing and fabrication techniques. Both well‐established micropatterned interfaces and the latest advancements in multidimensional hydrogel‐based structures are discussed. Finally, cutting‐edge clinical applications of bioelectronic systems that incorporate PEDOT:PSS are discussed, underscoring their potential in next‐generation medical technologies. Overall, this work presents a balanced and forward‐looking perspective that connects the evolution of PEDOT:PSS to its emerging role in clinically translatable bioelectronic systems.

## Graphical abstract

PEDOT:PSS ‐ Poly(3,4‐ethylenedioxythiophene) polystyrene sulfonate ‐ is typically processed from water dispersions to form multifunctional and multidimensional constructs with tunable electronic and ionic conductivity. Throught processing engineering, PEDOT:PSS is intergrated in bioelectronic devices that operate efficiently in physiological conditions and monitor and stimulate human tissue with unprecedented precision.

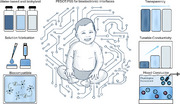

## 1 Introduction

Bioelectronic technologies are rapidly positioning themselves as key tools in modern healthcare. Devices, such as pacemakers and functional electrical stimulators, have been widely used in medical practice since the 1950s, laying the groundwork for today's more advanced, tissue‐integrated systems.^[^ ^1^ ^]^ Traditional electronic materials, such as silicon‐based semiconductors and metals, have propelled bioelectronic technologies to significant therapeutic breakthroughs. For example, the recent advancements in complementary metal‐oxide semiconductor (CMOS) electronics and artificial intelligence have enabled the development of implantable, miniaturized bioelectronic interfaces that can perform brain‐like computations within the body.^[^ ^2^ ^]^ In parallel, advancements on soft bioelectronic materials that adapt to natural movements have enabled seamless bioelectronic‐tissue interfaces that record/monitor biological activity with high precision and minimal damage.^[^ ^3^ ^]^

The conducting polymer blend PEDOT:PSS‐poly(3,4‐ethylenedioxythiophene) poly(styrenesulfonate ‐ stands out as a key material for bioelectronic interfaces. PEDOT:PSS belongs to the family of organic electronic materials—one of the few classes of materials that can enable both ionic and electronic charge transport simultaneously.^[^ ^4^ ^]^ This mixed ionic‐electronic conductivity is of paramount importance for bioelectronic interfaces. On one hand, the human body is made of soft organs, tissues, and cells orchestrated by the finely tuned movement of hydrated ions and charged organic molecules—a phenomenon known as bioelectricity.^[^ ^5^, ^6^ ^]^ On the other hand, electronics require materials that support electronic transport with high fidelity. Therefore, materials that support the transport of both ions and electrons allow for the direct translation of biological information into an electronic readout within a confined space of a few nanometres.^[^ ^7^, ^8^ ^]^

PEDOT:PSS is typically processed from water dispersions to form thin bioelectronic interfaces with solid‐state electronic conductivities in the range of 0.1–4000 S cm^−1^,^[^ ^9^, ^10^ ^]^ with recent studies showcasing electronic conductivity of 15 143 S cm^−1^ by treating films with volatilizable trifluoromethanesulfonic acid and patterned by blade coating to promote ordered stacking and crystallization of PEDOT chains.^[^ ^11^ ^]^ The ionic conductivity of PEDOT:PSS is typically relatively high, comparable to ion mobilities found in pure electrolytes, as reported by Stavrinidou et al. by using the electrochromic moving front experiments. This setup allows for the reliable extraction of ion mobility in PEDOT:PSS thin films, which was found in the order of 1 × 10^−4^ to 1 × 10^−3^ cm^−2^ V^−1^s^−1^.^[^ ^12^, ^13^ ^]^ Importantly, Rivnay et al. showed that the ion mobility of PEDOT:PSS films can be fine‐tuned with thin film microstructure control.^[^ ^7^ ^]^

The ionic conductivity/mobility in PEDOT:PSS thin films strongly depends on the electrolyte composition, concentration, and eventually the chemical environment of the cell/tissue media of the bioelectronic application of interest. Typical electrolytes include 0.1 M Sodium Chloride (NaCl),^[^ ^14^ ^]^ 0.1 M Potassium Chloride (KCl),^[^ ^15^ ^]^ and 10 mM Phosphate Buffered Saline (PBS).^[^ ^16^ ^]^ Therefore, tuning ionic transport in PEDOT:PSS films and constructs according to the bio‐application is an interesting gap to address. In general, through tailored processing, as it will be discussed in detail in the following sections, PEDOT:PSS has shown excellent operational stability in contact with aqueous/human tissue environments of diverse compositions. A prime example is shown in the work of Orlemann et al., where PEDOT:PSS electrodes remained stable for over 16 weeks of continuous stimulation in PBS, totaling over 10 billion stimulation pulses.^[^ ^17^ ^]^ An early 2011 study from Venkatraman et al. assessed PEDOT:PSS‐coatings by accelerated aging at 67 °C, causing changes after 4 weeks (∼32 weeks physiologically), due to delamination.^[^ ^18^, ^19^ ^]^ However, further investigation into the failure mechanisms of PEDOT:PSS‐based bioelectronics would greatly contribute to the design of robust, long‐term implantable devices. In particular, understanding and mitigating PEDOT:PSS thin‐film delamination from metal substrates is a key aspect that warrants detailed study.^[^ ^20^ ^]^ Strategies to enhance the stability include integrating them with other materials. For example, Blau et al., developed PEDOT:PSS‐b‐PPEGMEMA electrodes that were stressed with high‐frequency biphasic current pulses (500 µA, 100 µs, 50 Hz) and withstood approximately 1 billion pulses, making them highly suitable for long‐term recordings.^[^ ^21^ ^]^ While other emerging carbon‐based materials, such as graphene or carbon nanotubes (CNTs), are also being explored as candidates for electronic components, PEDOT:PSS has distinct features that promote it as a key material for emerging bioelectronic technologies. For example, CNTs provide excellent electrical and mechanical properties, yet concerns about their potential toxicity remain a major barrier for clinical use.^[^ ^22^ ^]^ Graphene, with its high electrical conductivity and biocompatibility, also shows promise for future bioelectronic applications. Challenges like complex fabrication steps and integration with conventional electronics currently limit its practical integration,^[^ ^23^ ^]^ with global efforts focusing on addressing these. As it is discussed in detail in the following sections, PEDOT:PSS combines strong substrate adhesion, reliable operation in aqueous electrolytes, and tunable electrical properties.^[^ ^24^, ^25^ ^]^ Additionally, compared with other organic electronic materials, such as p‐type glycolated thiophenes (e.g., p(g2T‐TT))^[^ ^26^, ^27^ ^]^ and n‐type ladder polymers (e.g., BBL,^[^ ^28^ ^]^ BBL:PEI^[^ ^29^ ^]^), PEDOT:PSS offers clear advantages. Many of these alternative polymers require organic solvents for processing, whereas PEDOT:PSS can be easily processed in water, making it highly biocompatible and practical to mix with water‐based materials that are present in the human body. Importantly for bioelectronics and the translation of these technologies in the clinic, PEDOT:PSS is the only organic electronic material that has been approved by the Food and Drug Administration (FDA) and the CE mark for its use in human clinical trials.^[^ ^30^ ^]^ Nevertheless, the development of these new organic electronic material systems is a highly promising field of research and breakthroughs in the clinical translation of these systems are expected in the coming years.

Here, we present an overview of the conductive polymer PEDOT:PSS, bridging its chemistry with its unique potential to be used in the clinic. We start with a historical investigation of how PEDOT:PSS emerged, followed by laying out its operation principles, and why these are almost unique for bioelectronic interfaces. We then focus on how water‐based PEDOT:PSS formulations can be controlled to fabricate multidimensional bioelectronic structures, spanning from ultra‐thin, micron‐sized electrodes to highly biomimetic, 3D structures. Finally, we review the state‐of‐the‐art bioelectronic devices and their potential to be used to improve the quality of life in patients with numerous conditions. This review builds on top of several extensive reviews over the last few years. Organic bioelectronic technologies have been reviewed by Berggren and Richter‐Dahlfors,^[^ ^31^ ^]^ Rivnay et al.,^[^ ^32^ ^]^ Simon et al.,^[^ ^33^ ^]^ and Pitsalidis et al.^[^ ^34^ ^]^ More specifically, PEDOT:PSS operation principles were reviewed by Crispin et al.,^[^ ^35^ ^]^ Donahue et al.,^[^ ^36^ ^]^ and Gueye et al.^[^ ^37^ ^]^ A broader review on conducting polymers and their interaction with biological systems was conducted by Zeglio et al.^[^ ^38^ ^]^ Other closely relevant reviews report the development of organic bioelectronic devices that are based on PEDOT:PSS,^[^ ^30^ ^]^ other organic mixed ionic‐electronic conductors,^[^ ^4^ ^]^ and organic electrochemical transistors (OECTs).^[^ ^39^, ^40^ ^]^ This report integrates insights from all these prior studies to deliver a comprehensive overview—from the historical progression of PEDOT:PSS to its emerging potential relevant to bioelectronic applications. We emphasize the extensive global efforts dedicated to elucidating the operational mechanisms associated with material processing, while examining recent advancements in PEDOT:PSS architectures, including electrically active hydrogel systems promising for future clinical applications.

## 2 Tracing the Evolution of PEDOT:PSS: A Historical Journey

**Figure 1:**
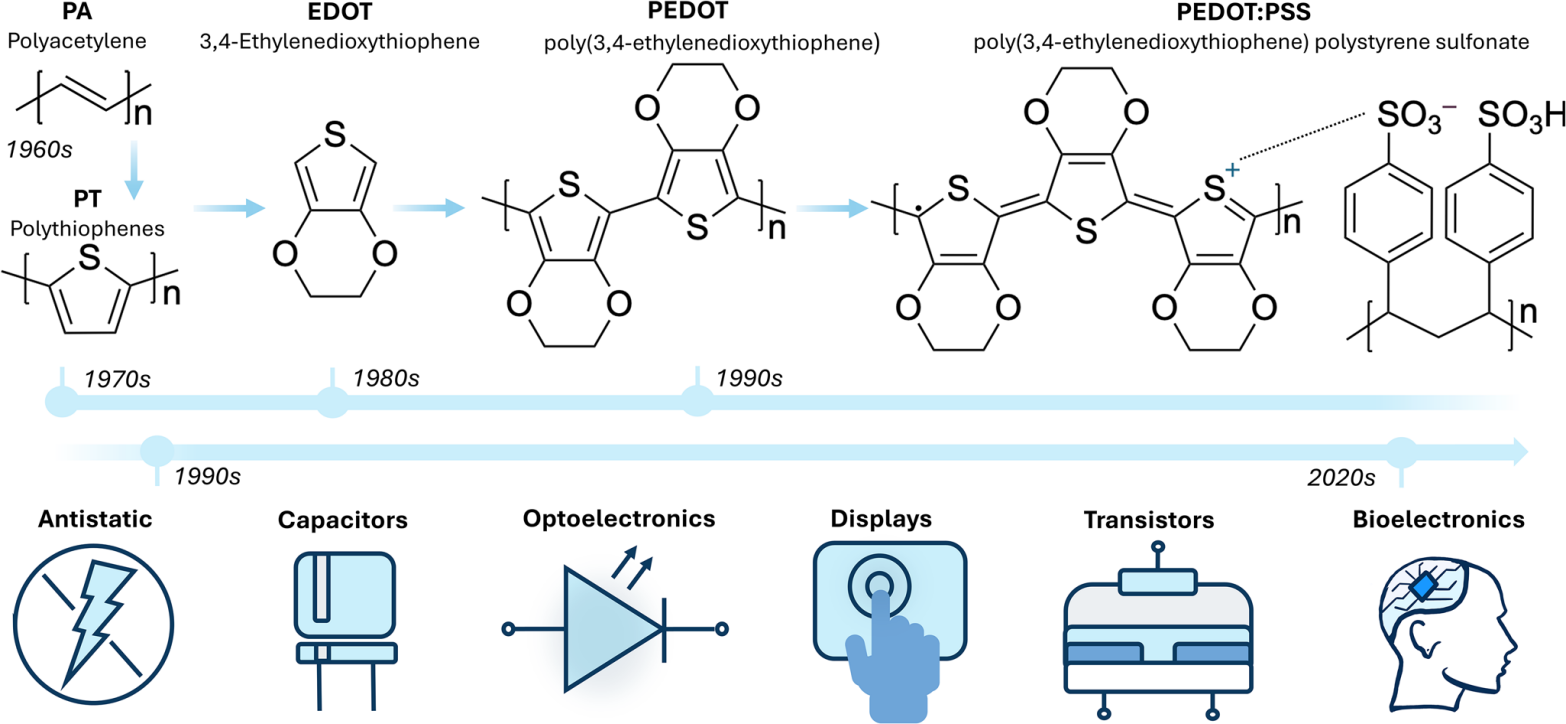
The development of PEDOT:PSS represents a major milestone in the field of conducting polymer chemistry and the development of organic electronics. Its origins trace back to the early discovery of polyacetylene. In the following years, extensive research into polythiophenes further expanded the understanding of charge transport in organic materials. From these foundations, PEDOT:PSS was developed as a water‐dispersible conductive polymer system. Originally developed as an antistatic coating, PEDOT:PSS has become essential in technologies such as capacitors, optoelectronic devices, electrochromic displays, and organic electronic components like transistors, and more recently, its biocompatibility and mixed ionic–electronic conductivity have made it a key material for bioelectronic interfaces.

PEDOT:PSS development was based on a series of breakthroughs in organic chemistry and materials science. The published work by S.C. Rasmussen on the historical evolution of conducting polymers related the origins even as far as 150 years ago.^[^ ^41^ ^]^ The chemical journey of PEDOT:PSS, as well as the key applications of the material, are summarized in **Figure** **1**. One of the first important milestones of this journey is the groundbreaking work from the Nobel laureates Karl Ziegler and Giulio Natta in Chemistry in 1963, for the development of catalysts enabling controlled olefin polymerization, which laid the foundation for exploring the polymerization of acetylene, marking a pivotal moment in the development of conducting polymers.^[^ ^42^, ^43^ ^]^ Hideki Shirakawa, Alan G. MacDiarmid, and Alan J. Heeger expanded on the synthesis and characterization of conducting polyacetylene^[^ ^44^, ^45^ ^]^ and showed that its electrical resistance can be tuned from insulating to metallic.^[^ ^46^, ^47^ ^]^ In this context, insulating refers to undoped conducting polymers, which have very high resistance because their electrons are localized and cannot move freely, while metallic refers to doped conducting polymers, where the introduction of charge carriers allows electrons (or holes) to move through the conjugated backbone, giving it low resistance, comparable to metals.^[^ ^48^, ^49^ ^]^ These pioneering studies established the concept of electronic conductivity in polymers, paving the way for the evolution of organic electronics and earning the Nobel Prize in Chemistry in 2000.^[^ ^50^ ^]^

The 1970s and 80s saw an increasing interest in this new class of materials, with established laboratories (e.g., IBM) interested and expanding towards electrochemical polymerization of conducting polymers.^[^ ^51^, ^52^, ^53^, ^54^ ^]^ Through this investigation, polythiophenes emerged as a model conducting polymer system.^[^ ^33^, ^34^, ^55^ ^]^ In 1988, researchers at Bayer AG's laboratories in Germany, filed a European patent (i.e., DE3813589 A1) for the development of certain derivatives of polythiophenes, and oxidative polymerization,^[^ ^56^ ^]^ followed by a patent disclosing the use of polythiophenes in capacitors (i.e., DE 3 814 730 A1), setting a precedent for applications in organic electronics.^[^ ^57^ ^]^ However, polythiophene was insoluble in common solvents and not compatible with the concept of solution‐processed electronics. To overcome this limitation, many substituted derivatives have been developed, with PEDOT emerging as a better choice due to its superior conductivity and stability.^[^ ^48^ ^]^ In 1991, the polymerization from 3,4‐ethylenedioxythiophene (EDOT) to poly(3,4‐ethylenedioxythiophene (PEDOT) was published.^[^ ^58^, ^59^ ^]^ The electrochemical stability of PEDOT immediately sparked interest in applications such as batteries and capacitors.^[^ ^60^ ^]^ While PEDOT (at that time named Baytron M, where M stands for monomer) provided significant control over physical and electronic properties, challenges related to its solubility and stability were still not addressed. The introduction of water‐soluble polyelectrolytes marked a breakthrough in overcoming this challenge. In 1995, Jonas combined PEDOT with poly(styrene sulfonic acid) (PSS)—a water‐soluble polyelectrolyte—to create a water‐processable PEDOT:PSS dispersion, which found its first application as an effective antistatic coating for photographic films.^[^ ^61^ ^]^

Fast forward to date, current PEDOT:PSS synthesis begins with the preparation of aqueous solutions of poly(styrene sulfonic acid) (PSS), an oxidizing agent, such as sodium persulfate (Na_2_S_2_O_8_), and a catalyst, such as iron(III) sulfate (Fe_2_(SO_4_)_3_). The liquid EDOT monomer is first added to the aqueous PSS solution. Subsequently, the oxidizing agent and catalyst solutions are introduced, initiating the polymerization reaction. The oxidizing agent initiates the oxidation of EDOT, resulting in the formation of EDOT radical cations that polymerize into PEDOT chains. EDOT, as a monomer, cannot conduct electricity or form stable films on its own. Polymerizing it into PEDOT gives it intrinsic conductivity, and adding PSS (PEDOT:PSS) makes it water‐dispersible, processable, mechanically stable, and suitable for bioelectronic applications. Concurrently, the negatively charged PSS^−^ chains interact with the positively charged PEDOT chains, resulting in a core‐shell nanostructure of stable PEDOT:PSS colloidal particles in water with balanced charge. While the addition of PSS^−^ improves solubility and film formation, the ratio of PEDOT to PSS can be adjusted during synthesis to tune the electrical properties of the material.^[^ ^62^, ^63^ ^]^ This combination produces a water‐soluble polyelectrolyte system with excellent transparency and stability and can be manipulated at ambient conditions with thermal treatments and processing additives. Initially marketed as Baytron P by H.C. Starck GmbH (now CLEVIOS™), PEDOT:PSS is to date one of the most popular and reliable commercially available p‐type conducting polymers, and can be purchased in various formulations tailored for a number of (opto)electronic applications.^[^ ^64^ ^]^ The commercialization of stable PEDOT:PSS water dispersions triggered a surge of applications, establishing PEDOT:PSS as a key material in organic electronics. PEDOT:PSS drove the development of organic optoelectronic devices, such as organic light‐emitting diodes (OLEDs)^[^ ^65^ ^]^ and organic photovoltaics (OPVs),^[^ ^66^ ^]^ where it serves as the hole injection/transport layer. Continuous breakthroughs and extensive research advanced a broad range of applications, including supercapacitors,^[^ ^67^ ^]^ transistors,^[^ ^39^ ^]^ as well as touchscreens and flexible displays^[^ ^68^ ^]^ that are used in smartphones and screens commercialized by leading brands. This extensive research on PEDOT:PSS formulations and their applicability in organic electronic devices has provided valuable lessons that have greatly benefited the development of organic bioelectronic devices, which will be discussed in the next section.

## 3 From Thin Films to 3D Constructs: Functional PEDOT:PSS Bioelectronic Interfaces

PEDOT:PSS constructs possess a unique combination of properties that make them ideal for direct, functional interfacing with living cells and tissues. These properties can be fine‐tuned through processing and tailored for the bioelectronic application of interest. This section provides an overview of how PEDOT:PSS water‐based dispersions are transformed into functional constructs of different dimensions and architectures, and support simultaneous transport of ions and electrons. The possibility to mix PEDOT:PSS with other water‐soluble components of the biological milieu allows for the development of truly biomimetic interfaces that bridge the electrical, chemical, and mechanical mismatch between biological and electronic systems.

### 3.1 From Nano‐Dispersions in Water to Functional Thin Films that Conduct Ions and Electrons

PEDOT:PSS typically comes in water dispersions of particles between 20  and 200 nm in diameter.^[^ ^69^ ^]^ As also discussed in section 2, such dispersions are made of two distinct polymers: PEDOT and PSS. Positive charges in PEDOT backbone (10–20 repeating units) are compensated by negative charges provided by the macromolecular polyelectrolyte PSS (more than a thousand repeating units),^[^ ^70^ ^]^ forming a stable colloidal particle dispersion in water. The colloidal stability of PEDOT:PSS dispersions arises from the extended PSS chains surrounding the PEDOT‐rich cores, which impart high surface charge and stabilize the complex in dispersion.^[^ ^69^ ^]^

**Figure 2:**
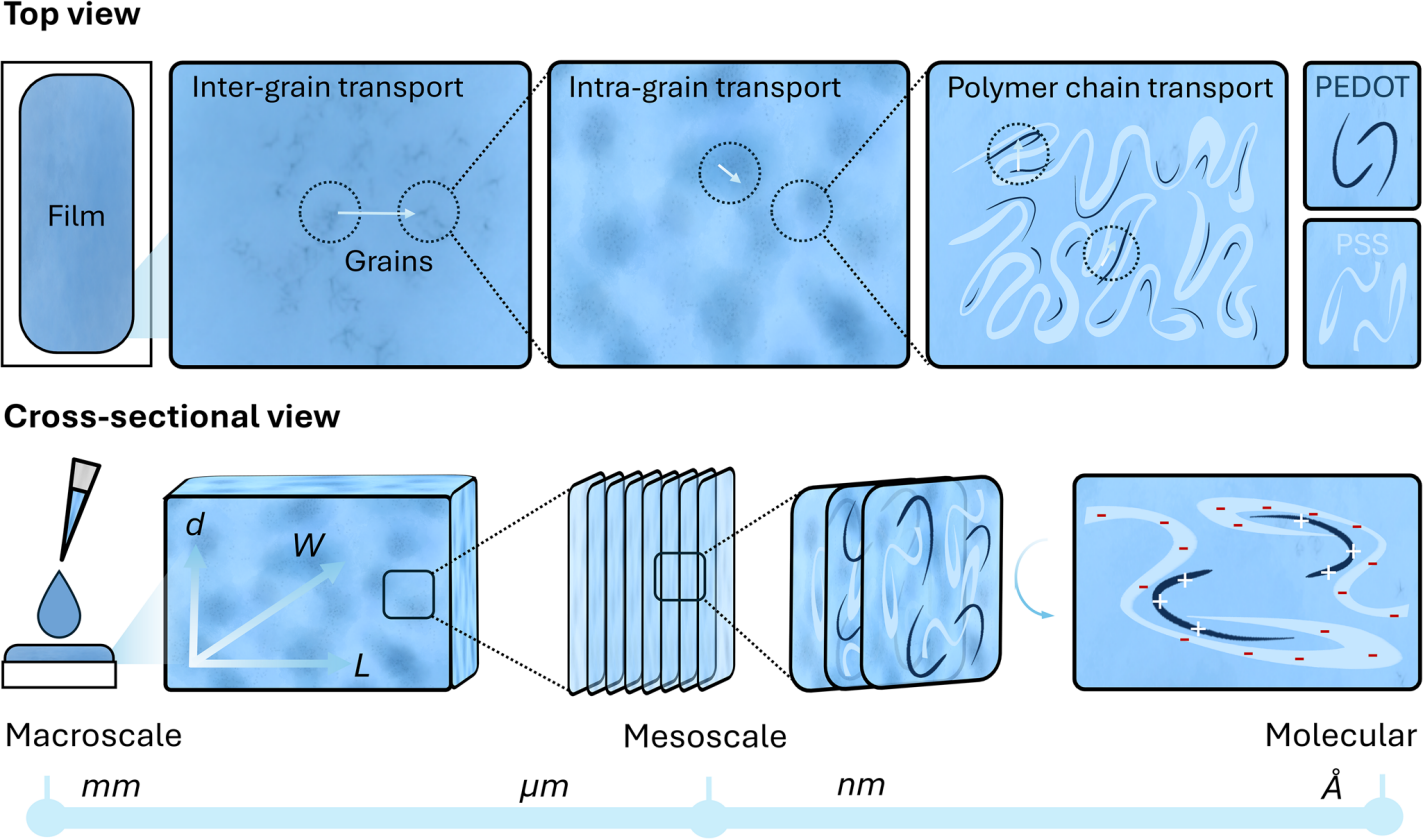
PEDOT:PSS thin film structure in the macroscale, mesoscale, and molecular scale. The top panel represents a top view of the film's microstructure and morphology. In the macroscale, the films transport electronic charge between neighboring PEDOT‐rich clusters (grains; inter‐grain transport). In the mesoscale, intra‐grain transport takes place between PEDOT grains, via electronic charge transport within the PEDOT backbone chains, which are separated by distinct PSS regions. The bottom panel represents a side view of the thin film, which can be observed at the microscale as a bulk or 3D pattern. Ionic transport can occur throughout this volumetric structure, characterized by a length 𝐿, width 𝑊, and thickness 𝑑 (ranging from ∼50 nm up to several microns). Individual polymer backbones can stack on top of each other, forming parallel tiny capacitors created by ions and electronic carriers (holes) throughout the film volume.

When PEDOT:PSS is cast to form a thin film, two distinct phases form: PEDOT:PSS‐rich regions and PSS‐rich regions.^[^ ^35^ ^]^ As the hydrophilic PSS is in excess, the deposition and drying of the PEDOT:PSS film result in a pancake‐like morphology of grains with a PEDOT‐rich core and a PSS‐rich shell. As a result, the thin film morphology of PEDOT:PSS is comprised of closely packed PEDOT‐rich domains in a PSS‐rich matrix. The PEDOT‐rich regions are highly conductive, whereas the PSS‐rich regions are mostly insulating, yet allow for efficient electron and hydrated ion transport.^[^ ^8^ ^]^ Hydrated ions traverse along the hydrophilic PSS chains within the PEDOT:PSS construct through a solvated/vehicle mechanism, with ionic diffusion constants comparable to those of the pure liquid phase.^[^ ^13^, ^71^ ^]^ Therefore, a balance between ionic and electronic conductivity can be achieved by optimizing the microstructure of the film.^[^ ^72^ ^]^ PEDOT:PSS mixed conductivity is strongly dependent on film morphology in the macro‐, meso‐, and molecular scale and can be tuned through processing, solvent engineering,^[^ ^8^ ^]^ dopants,^[^ ^73^ ^]^ nanocomposites,^[^ ^74^ ^]^ and post‐treatments.^[^ ^75^ ^]^ As schematically depicted in **Figure** **2**, the polymer forms a two‐phase system where conducting PEDOT‐rich domains, or “grains,” are embedded in insulating PSS. Electronic charge transport occurs both within grains (intra‐grain) and between grains (inter‐grain). Intra‐grain transport involves conduction along the conjugated PEDOT backbones, which is primarily determined by the conjugation length and π–π stacking distance between PEDOT chains. Stronger and denser π–π interactions result in more efficient charge transfer.^[^ ^76^ ^]^ Inter‐grain transport depends on the size, elongation, and connectivity of PEDOT grains.^[^ ^8^ ^]^ Therefore, both intra‐ and inter‐grain pathways must be optimized to maximize overall conductivity. Hence, understanding charge transport at multiple scales is essential to maximize ionic and electronic conduction. The concept of optimal mixed ionic and electronic conductivity is reflected by the product of volumetric capacitance (C*) multiplied by the electronic mobility (*µ*). C* quantifies how much charge a material can store per unit volume, while *µ* is a measure of how quickly charge carriers (holes or electrons) can move through a material under an electric field. Proctor et al. developed a model for the ideal C* in PEDOT:PSS, where it is suggested that the capacitance increases linearly with the volume of the film, not just its area. This means that charge is stored throughout the entire bulk of the material. Each ion that enters the film replaces a hole that leaves, forming tiny local double‐layer capacitors. In simple terms, the model describes the polymer as a three‐dimensional lattice of these microscopic capacitors, spaced about 1.8 nm apart. Each layer of sites behaves like a capacitor plate, and all the layers together act as many capacitors connected in parallel, producing a total capacitance that reflects the sum of all these layers within the volume. The closer the sites are to each other, the higher the overall capacitance of the film.^[^ ^77^ ^]^ These theoretical calculations for PEDOT:PSS resulted in a C* value of 39 Fcm^−3^, a number that was also verified experimentally by Rivnay et al.^[^ ^78^ ^]^ Several operando techniques have been developed to characterize the charging/discharging of PEDOT:PSS, providing useful insights into the mechanisms of mixed charge transport.^[^ ^79^, ^80^ ^]^ Moreover, such studies highlighted the importance of interface engineering at the polymer/metal interfaces to modulate ion transport and eliminate unwanted faradaic reactions, respectively.^[^ ^81^ ^]^

**Figure 3:**
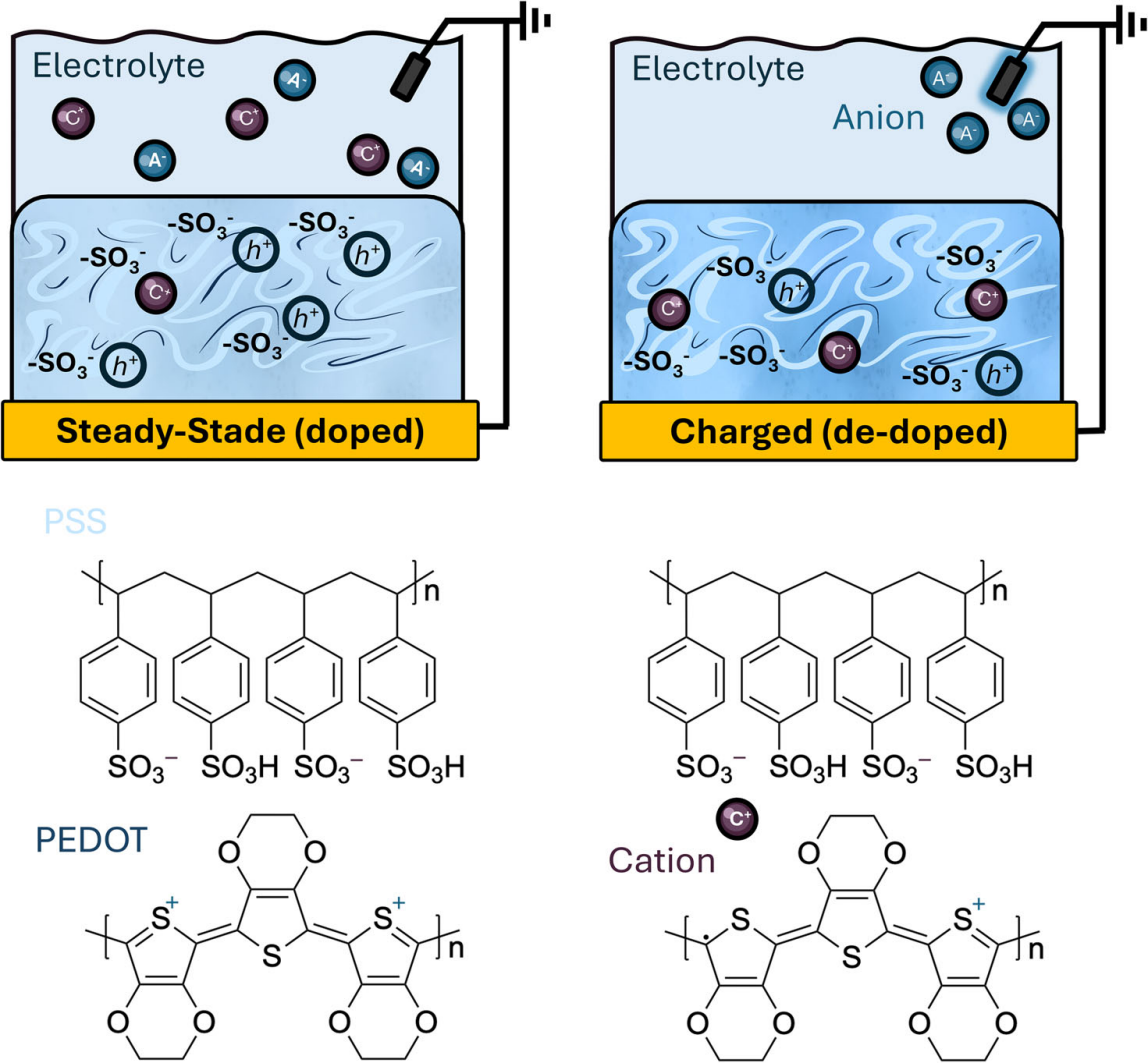
A schematic representation of the charging process of PEDOT:PSS thin films coated over a conducting substrate (e.g., gold) in cross‐sectional view, showing the interactions with both hydrated anions and cations. Under steady‐state conditions with no applied bias, the film is in a relatively neutral state, exhibiting high electronic conductivity, while ions are passively absorbed into the film. When a bias is applied, the film is charged: cations from the electrolyte compensate for the PSS^−^ anions, and de‐dope PEDOT:PSS (PEDOT + Cation⁺:PSS^−^). This de‐doping process reduces the electronic conductivity of the film. This charging process of PEDOT:PSS films also results in distinct changes in color, a property known as electrochromism. The ability to reversibly switch PEDOT:PSS from a conductor to a more insulating state makes it particularly useful for switching applications in bioelectronics.

To further elaborate on the charging mechanisms of PEDOT:PSS films, we can look at it from the electrochemical doping/dedoping perspective. PEDOT:PSS is usually deposited on top of metal contacts (e.g., gold) and immersed in electrolytes with supporting reference and counter electrodes, as shown in **Figure** **3**. Since PEDOT:PSS is already heavily doped with PSS^−^, further doping with negatively charged ions (anions such as Cl^−^) results in subtle changes in PEDOT:PSS thin film conductivity and capacitance.^[^ ^82^ ^]^ In contrast, PEDOT:PSS films can be fully de‐doped with positively charged ions (cations, such as Na^+^) with a dramatic decrease in thin film conductivity and an increase in the film capacitance. In this case, the ionic bonds of PEDOT^+^:PSS^−^ break and cations from solution compensate for the negative charges in PSS^−^, forming a new ionic bond between cation^+^:PSS^−^. This mechanism takes place all over the volume of the film, as explained by the Proctor model mentioned in the previous paragraph, and majorly contributes to the high volumetric capacitance of PEDOT:PSS under constant bias. This reduction in conductivity and increase in capacitance provide an effective and reversible way to modulate the film´s conductivity over multiple cycles, a property that is fundamental to the operation of PEDOT:PSS electrodes for cell recording and stimulation, as well as organic electrochemical transistors.^[^ ^83^ ^]^ As it is described in the next sections, this fully reversible electrochemical process and can be fine‐tuned via fabrication and material's processing engineering.

### 3.2 Microfabricated PEDOT:PSS constructs—1D and 2D

As mentioned in Section 3.1, the ability to control the mixed ionic–electronic conduction of PEDOT:PSS through processing enables precise tuning of its properties, providing opportunities to optimize PEDOT:PSS for various bioelectronic applications. Importantly, processing approaches are highly compatible with microfabrication and lithographic patterning in cleanroom settings,^[^ ^84^ ^]^ and allow for the precise fabrication of bioelectronic devices such as microelectrode arrays. This is difficult with silicon‐based bioelectronics, which require complex fabrication to develop functional bioelectronic interfaces. In the following paragraphs, we describe the different processing approaches that are used to make functional PEDOT:PSS films and their integration in device architectures.

The inclusion of processing additives in the water‐based PEDOT:PSS dispersion has an impact on film morphology and microstructure, and hence modifies the optoelectronic properties of the film. Polar solvents with high boiling point, such as dimethyl sulfoxide (DMSO) or ethylene glycol, are typically used for this purpose. The inclusion of these processing additives induces a liquid phase separation of PEDOT and PSS, resulting in a more compact, conductive PEDOT‐rich film after slow drying at room temperature conditions.^[^ ^85^, ^86^, ^87^ ^]^ Importantly, as shown by Rivnay et al., both the ionic and hole conductivities of PEDOT:PSS can be fine‐tuned and maximized by modifying the ethylene glycol content.^[^ ^88^ ^]^ Dodecylbenzenesulfonic acid (DBSA) is also a common processing additive that is used to facilitate film processing of PEDOT:PSS water dispersions.^[^ ^89^ ^]^ Zhang et al. found that, by keeping DBSA concentration at 0.5 v/v %, homogeneous films over larger areas can be achieved—an important aspect for reliable microfabrication of PEDOT:PSS bioelectronic interfaces – and film conductivity up to 500 S cm^−1^. However, DBSA concentrations above ∼0.5 v/v %, in addition to decrease the surface tension, induce phase separation, hindering film uniformity after spin‐coating.^[^ ^90^ ^]^

To complement these conductivity improvements, additional additives are required to render the films stable in water and promote long‐term stable device operation in contact with aqueous media. Crosslinkers, such as (3‐glycidyloxypropyl)trimethoxysilane (GOPS), are important for this purpose. These additives are used to prevent film solubility in water and substrate delamination and have been extensively used to stabilize PEDOT:PSS films for bioelectronic interfaces. The crosslinking reaction is initiated by temperature (typically above 100 °C, protocols can go to 140°C for 30 min) and creates strong GOPS‐PSS, GOPS‐GOPS, and GOPS‐substrate bonds, rendering the film insoluble in water and stably adherent on substrates like glass, gold, and other metals.^[^ ^25^ ^]^ However, this comes at the cost of reduced ion mobility, due to the presence of phase‐segregated, siloxane‐rich domains that are less polar and hinder the transport of hydrated ions.^[^ ^91^ ^]^ The use of polyethylene glycol diglycidyl ether (PEGDE) as the crosslinker mitigates conductivity issues as presented in GOPS. The absence of silane groups in the PEGDE moiety helps to maintain high ionic and electronic conductivities in PEDOT:PSS, yet the adhesion on metal substrates is weak.^[^ ^92^ ^]^ In addition, several adhesion‐promoting layers have been introduced to provide strong adhesion without compromising electrical properties and biocompatibility. Examples include polyurethane on glass and silicon,^[^ ^93^ ^]^ polydopamine primers on poly(propylene) and poly(ethylene terephtalate),^[^ ^94^ ^]^ and diazonium salts grafting on platinum.^[^ ^95^ ^]^

Often, the use of processing additives and crosslinkers is complemented by post‐processing steps to maximize mixed conduction through microstructure changes and to promote stability in water electrolytes and tissue environment. Thermal treatment (commonly known as thermal annealing) is an effective method for post‐processing films. It involves exposing the films at temperatures in the range between 40–180 °C to evaporate remaining solvents, increase chain mobility towards a state of thermodynamic equilibrium (i.e., better chain packing), and to promote the adhesion of the conducting polymer to the substrate.^[^ ^96^ ^]^ A recent example by Doshi et al., shows that a short annealing of 2 min at 150 °C yields functional PEDOT:PSS films with improved conductivity and C*, without the need of external crosslinking additives.^[^ ^75^ ^]^

All the processing strategies described above allow for the facile processing of functional PEDOT:PSS films and are combined with established microfabrication techniques in cleanroom settings. PEDOT:PSS is compatible with photolithography, etching, and chemical vapor deposition—techniques that are commonly used in the cleanroom to fabricate high‐resolution devices, such as microelectrode arrays (MEAs). A detailed review of the microfabrication of PEDOT:PSS‐based devices has recently been reported by Z. Lu et al.^[^ ^84^ ^]^ Solution deposition techniques, such as spin‐coating,^[^ ^97^, ^98^ ^]^ spray‐coating,^[^ ^99^ ^]^ and doctor blading^[^ ^100^ ^]^ are commonly used to deposit functional PEDOT:PSS thin films. Post‐deposition patterning of PEDOT:PSS thin films can also be achieved via etching or direct laser patterning, to either remove the material or decrease the conductivity of the film in specific areas.^[^ ^101^, ^102^ ^]^ Given the polymeric nature of PEDOT:PSS, devices can be fabricated on a variety of substrates, paving the way for soft and conformable bioelectronics.^[^ ^103^ ^]^ Flexible substrates such as Parylene C,^[^ ^104^, ^105^, ^106^ ^]^ polydimethylsiloxane (PDMS),^[^ ^107^ ^]^ and polyimide,^[^ ^108^ ^]^ with and without metal tracings^[^ ^109^ ^]^ have been used to deposit PEDOT:PSS micropatterns and to develop precise, highly biomimetic bioelectronic interfaces.

Another promising way for the selective patterning of functional PEDOT:PSS films is electrochemical polymerization (or electropolymerization).^[^ ^110^ ^]^ This technique does not involve commercially available water dispersions of PEDOT:PSS, but is based on the electropolymerization of the EDOT monomers. The polymerization commences with the preparation of an electrolyte solution, wherein EDOT monomers are dissolved alongside poly(sodium 4‐styrenesulfonate)  (PSS). Subsequently, a three‐electrode electrochemical cell is used, comprising a working electrode, which serves as the substrate for PEDOT:PSS growth (e.g., gold), a counter electrode, typically a platinum wire, and a reference electrode, typically Ag/AgCl. The polymerization reaction is initiated at the working electrode surface, where PEDOT chains begin to form,^[^ ^111^, ^112^ ^]^ producing a thin film with controlled thickness and morphology that can stably operate in electrolytes and tissue environments without requiring a crosslinker. Electrochemical deposition relies on polymer growth and decreasing solubility; if solubility remains high, film formation is poor. Additionally, as film thickness increases, adhesion weakens, limiting long‐term stability.^[^ ^113^ ^]^

Finally, inkjet‐printing technologies can also be used to deposit 1‐dimensional, functional PEDOT:PSS constructs, enabled by drop‐on‐demand deposition. For example, inkjet‐printed PEDOT:PSS lines have been used to create precise multi‐electrode arrays for in vitro electrophysiology^[^ ^114^ ^]^ and paper‐based electrodes for electrocardiography.^[^ ^115^ ^]^ In some more examples, a PEDOT:PSS‐based polymer blend combined with polyethylene oxide (PEO), was patterned with inkjet printing and served as stretchable interconnects in wearable devices for health monitoring^[^ ^116^ ^]^ as well as MEAs for neuromuscular cartography.^[^ ^117^ ^]^

### 3.3 Multidimensional PEDOT:PSS‐Based Bioelectronics and Hydrogels—3D and 4D

The ability of PEDOT:PSS to be processed from aqueous dispersions offers unique opportunities for blending with biomolecules and extracellular matrix components, enabling the development of highly biomimetic electronic devices.^[^ ^118^ ^]^ These constructs can have tunable mechanical, electrical, and chemical properties and open up great possibilities for biohybrid electronic implants as well as for in vitro systems, as it is described in detail in Section 4.

Generally, the term “conducting polymer scaffolds” is used to define conducting, PEDOT:PSS‐based 3D constructs that integrate a macro‐porous structure, recapitulating the tissue and extracellular matrix architecture. These constructs are used to support cell adhesion and 3D tissue formation.^[^ ^119^, ^120^ ^]^ Lyophilization (freeze‐drying) and ice‐templating are the most commonly used methods to develop macro‐porous PEDOT:PSS scaffolds.^[^ ^121^, ^122^ ^]^ The pore size, architecture, and the mechanical and electrical properties of the scaffolds can be tuned by adjusting factors such as solution composition, freezing temperature, and drying rate. This allows for pore size distributions ranging from 10 to 200 µm.^[^ ^123^, ^124^, ^125^ ^]^ Although both methods rely on solvent sublimation, ice‐templating leverages the anisotropic growth of ice crystals to create aligned and tunable pore structures—something not possible with freeze drying.^[^ ^126^ ^]^ Moreover, combining ice‐templating with sulfuric acid crystallization enabled the formation of PEDOT:PSS scaffolds with mechanical and electrical anisotropy, offering advantages in controlling cell orientation and growth.^[^ ^127^ ^]^

Electrically active hydrogels based on PEDOT:PSS have recently gained significant attention as a promising platform for dynamic and tissue‐mimetic bioelectronics. Hydrogels, as defined by the International Union of Pure and Applied Chemistry (IUPAC), are “a gel in which the swelling agent is water”. This class of materials has made significant breakthroughs in bioengineering and has been used extensively in clinical applications. PEDOT:PSS‐based hydrogels combine the highly biocompatible nature of hydrogels with the electrical functionality of PEDOT:PSS, enabling biohybrid electronic constructs and devices that seamlessly integrate with biological systems.^[^ ^128^, ^129^ ^]^

Electrically active PEDOT:PSS hydrogels can be formed by three main routes: 1) physical blending PEDOT:PSS nanoparticles with hydrophilic polymers (e.g., gelatin, poly(vinyl alcohol) (PVA), and alginate), followed by crosslinking or gelation of the host polymer network;^[^ ^130^, ^131^, ^132^, ^133^ ^]^ 2) in situ polymerization of EDOT monomers within a pre‐formed hydrogel matrix;^[^ ^134^, ^135^, ^136^ ^]^ 3) “pure” PEDOT:PSS hydrogels by inducing the gelation with the use of additives, such as DMSO or DBSA.^[^ ^137^, ^138^, ^139^ ^]^ The route of forming PEDOT:PSS hydrogels defines the electrical and mechanical properties of the construct. Unlike PEDOT:PSS treated thin‐films, which can reach conductivities exceeding 10 000 S cm^−1^, PEDOT:PSS hydrogels typically exhibit lower values (0.001–50 S cm^−1^).^[^ ^134^ ^]^ For example, electrically active hydrogels made with route 1—i.e., PEDOT:PSS conducting particles embedded within an “inert” polymer gel network—typically show conductivities in the range of 0.001–1 S cm^−1^, as electron conductive pathways are minimized. However, this method offers the advantage of ultra‐soft hydrogels (i.e., G’ = 1–10 kPa), making it well‐suited to integrate cells and growing 3D soft tissues,^[^ ^140^, ^141^ ^]^ such as for injectable systems that can drive stem cell growth.^[^ ^142^ ^]^ PEDOT:PSS hydrogels made with route 2–i.e., electropolymerized PEDOT:PSS hydrogels that form interpenetrating networks with hydrophilic polymers—typically show higher electrical conductivity values compared to hydrogels made with route 1, due to the better intercalation of the conducting PEDOT:PSS pathways.^[^ ^136^, ^143^, ^144^ ^]^ In addition, the ability to electropolymerize hydrogels from EDOT monomers enables the selective patterning of soft electrodes to electrically interface the nervous system. Finally, PEDOT:PSS made with route 3—i.e., pure PEDOT:PSS hydrogels that can be formed by the use of additives—can achieve the highest electrical conductivity values due to the denser PEDOT:PSS network that support electronic transport. Sua ch denser network results in stiffer constructs with stretchable^[^ ^134^ ^]^ and self‐healing properties.^[^ ^143^ ^]^ This diversity in the properties of PEDOT:PSS hydrogels enables a wide range of bio‐interfacing and applications, as it is also discussed in Section 4. However, PEDOT:PSS‐based hydrogel systems represent a relatively new research area, and a key challenge lies in the fundamental understanding of the electronic and ionic conduction pathways within these materials. Although recent studies have provided valuable insights into these processes,^[^ ^145^ ^]^ the broad range of fabrication methods and the resulting diversity in material properties highlight the need for further systematic electrical and electrochemical characterization of such systems. In addition, for further integration of these promising systems in applications in bioelectronics, better fabrication and adhesion strategies between the “soft” hydrogel and “hard” substrates that are typically used (e.g., gold/glass) need to be developed.^[^ ^146^, ^147^ ^]^

Additive manufacturing is one of the most promising fabrication routes to integrate PEDOT:PSS‐based electrically conducting hydrogels in bioelectronic devices and constructs.^[^ ^148^ ^]^ 3D extrusion‐based printing enabled the fabrication of PEDOT:PSS hydrogels with resolution of ∼ 100 µm. For example, 3D‐printed PEDOT:PSS hydrogel scaffolds with physiologically relevant stiffness (<100 kPa) were developed and demonstrated to support cell growth, offering a customizable cell culture platform.^[^ ^149^ ^]^ In another study, X. Zhang et al. developed a conducting PEDOT:PSS–PVA–polyacrylic acid (PAA) bio‐adhesive hydrogel, achieving a relatively high printing resolution of approximately 60 µm, a high elongation at break (267%), low Young's modulus (∼12.79 kPa), electrical conductivity of 0.04 S cm^−1^, and strong adhesion on different substrates.^[^ ^150^ ^]^ Similarly, Rutz et al. reported an extrusion‐based 3D printed PEDOT:PSS‐based hydrogels with tissue‐mimicking properties.^[^ ^151^ ^]^ PEDOT:PSS‐based hydrogels can also be engineered for light‐based 3D printing by incorporating photopolymerizable linkers, enabling a high‐resolution honeycomb‐like structure with 500 µm hole size.^[^ ^152^ ^]^ High‐resolution patterning (i.e., 5 µm) and good adhesion on flexible substrates were also achieved by the design of PEDOT:PSS hydrogels with a light‐curable polymer matrix, leading to conductivity of ∼30 S cm^−1^ and tensile strain up to 50%.^[^ ^153^ ^]^ Finally, a laser method was reported to pattern gold nanoparticles‐loaded PEDOT:PSS hydrogel films. The authors took advantage of the strong electric field and photothermal energy of a continuous laser beam to induce phase separation of PEDOT:PSS and micropattern the hydrogel films down to 6‐µm in resolution, with constructs being electrochemically stable in PBS buffer for over 6 months. Plasmonic Au‐nanoparticles served to intensify the interaction of the laser with the PEDOT:PSS hydrogels. These constructs were used for both in vitro and in vivo studies in mice to stimulate the peripheral nervous system.^[^ ^154^ ^]^

**Figure 4:**
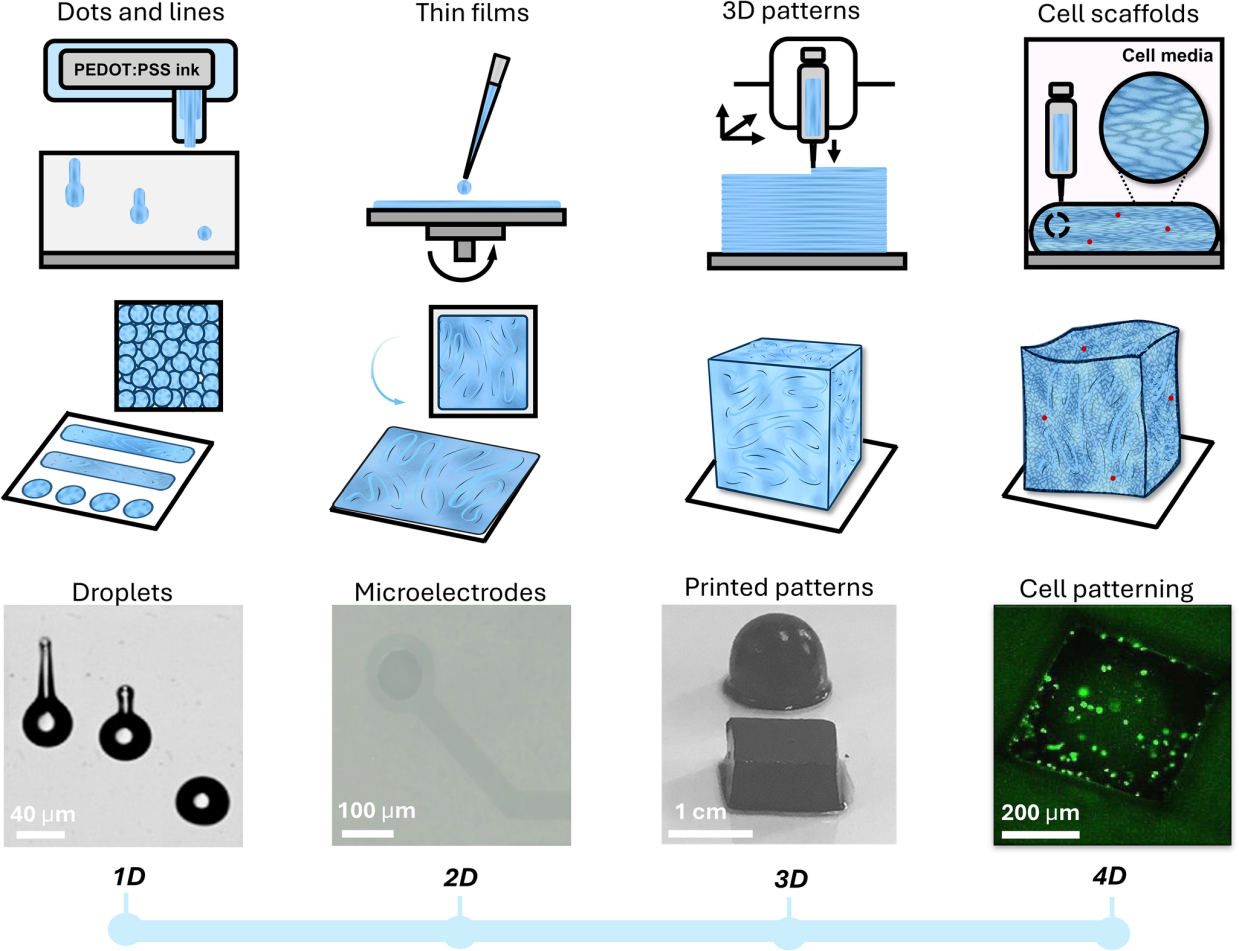
Multidimensional processing of PEDOT:PSS constructs, typically processed from water‐based dispersions. Biohybrid constructs with a diverse range of shapes and sizes, as well as electrical and mechanical properties, are possible. From left to right,1D, discrete dots are formed by tuned drop ejection with techniques such as inkjet printing – image adapted from^[^ ^161^ ^]^); 2D, microfabricated thin films from solution with several techniques such as spin coating. Thin films can be formed on a variety of substrates from transparent glass to flexible materials (an example of a film as part of a multielectrode array image adapted from^[^ ^162^ ^]^); Three‐dimensional architectures that can be created by 3D printing techniques such as extrusion printing—images of printed patterns adapted from^[^ ^163^ ^]^; and 4D can be used by techniques such as bioprinting in which the ink can be mixed with cells during or after extrusion within a dynamic, biohybrid ionic‐electronic system – a scaffold grid image with cells is shown as an example and adapted from.^[^ ^164^ ^]^

More dynamic patterns of PEDOT:PSS‐based constructs have also been demonstrated by the 4D approach.^[^ ^155^ ^]^ In this context, the “fourth dimension” refers to time, highlighting the construct's ability to transform within 3D fabrication. These interactions can trigger or guide the material's transformation, enabling dynamic responses in living bio‐systems.^[^ ^156^ ^]^ Furlani et al. reported composites of gelatin and PEDOT:PSS for implantation and proliferation of cardiomyoblasts.^[^ ^157^ ^]^ Choosing and designing suitable hydrogel systems for bioelectronics necessitates a comprehensive understanding of their physical, chemical, and biological properties, alongside a clear grasp of structure‐property relationships.^[^ ^158^ ^]^ Goding et al. developed a conducting hydrogel by covalently linking sulfonate groups to PVA macromers, enabling controlled dopant placement and improved physical, electrochemical, and mechanical properties.^[^ ^135^ ^]^ Overall, the ability of PEDOT:PSS to formulate biohybrid inks for additive manufacturing has already advanced soft, electrically active biomaterials structuring and shows potential for truly biomimetic electrical interfaces. Future research could explore even more dynamic 3D/4D structuring technologies such as volumetric light^[^ ^159^ ^]^ and acoustic^[^ ^160^ ^]^ printing, expanding the functional integration of dynamic, PEDOT:PSS‐based constructs in bioelectronics for clinical applications. An overview of the multidimensional constructs based on PEDOT:PSS, from 1D to 4D, is schematically presented in **Figure** **4**, coupled with representative examples from the literature.

## 4 Bioelectronic medicine based on PEDOT:PSS

**Figure 5:**
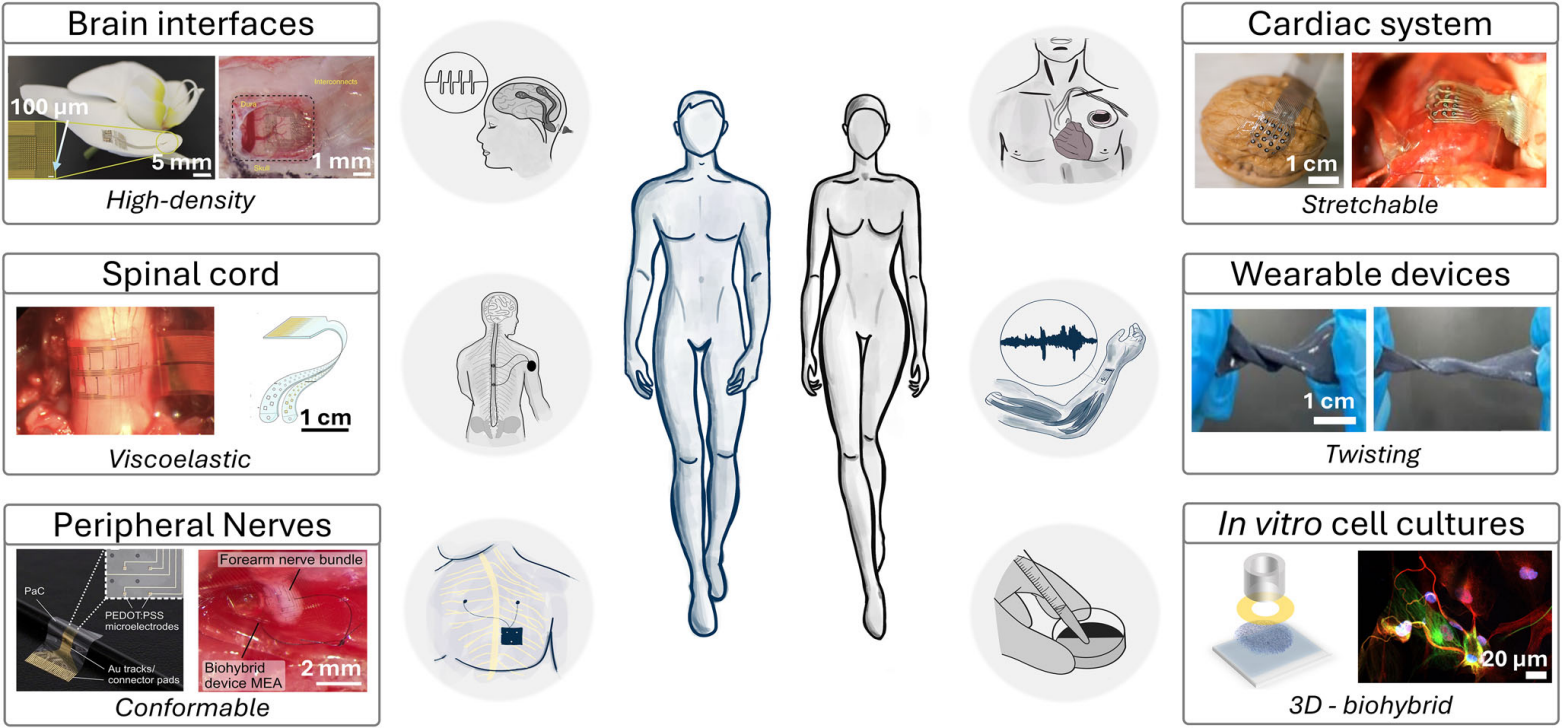
Schematic representation of bioelectronic medicine applications, highlighting the translational potential of PEDOT:PSS‐based bioelectronic interfaces. This figure presents both a general overview of various applications and examples of real devices reported in the literature. Applications include spinal cord and nervous system interfaces, in vitro studies, neural interfaces, cell recording (e.g., cardiac), and wearable devices. The figure demonstrates how PEDOT:PSS is versatile, flexible, biocompatible, and shows high potential for real bioelectronic applications. Figures and images from the literature are adapted with permission from refs. [106, 123, 177, 178, 179, 180].

Bioelectronic medicine is an emerging field focused on treating and diagnosing diseases using electrical signals.^[^ ^165^ ^]^ Microelectrode arrays (MEAs) are essential devices in this field, as they serve as the direct contact with tissues and, with the support of backend electronics, can both record and stimulate neural activity. Prominent examples are the UTAH array,^[^ ^166^ ^]^ the Michigan array,^[^ ^167^ ^]^ as well as flat metal arrays, intended for neural recording and stimulation. PEDOT:PSS is emerging as a key material for MEAs, as it helps to overcome limitations of existing technologies, such as the interface impedance—the resistance between the electronic implant and the human tissue^[^ ^168^ ^]^ and the conformability with human tissues.^[^ ^169^ ^]^ These properties are beneficial for both recording and stimulating electrodes, though the performance criteria differ. For recording applications, low impedance reduces thermal noise, leading to a higher signal‐to‐noise ratio (SNR) and improved recording resolution. As discussed in Section 2, the volumetric transport of both ions and electrons within PEDOT:PSS films (typically in the range of 100 nm) leads to high volumetric capacitance (C*) and low impedance, resulting in electrodes with high spatiotemporal resolution and high SNR.^[^ ^170^ ^]^ In contrast, stimulation electrodes are evaluated based on their ability to safely deliver charge, with charge injection capacity (CIC) being the figure‐of‐merit. The high capacitance of PEDOT:PSS allows for efficient charge delivery without reaching damaging voltage thresholds, making it suitable for safe and effective stimulation.^[^ ^171^ ^]^ Charge injection capacity for PEDOT:PSS MEAs is reported to be up to 9.5× compared to metal electrodes.^[^ ^172^ ^]^ For these reasons, a number of companies turned to PEDOT:PSS as a promising solution for pushing bioelectronic medicine to the clinic. Amplicoat (proprietary of this conducting polymer coating) achieved FDA approval in one application in 2016^[^ ^30^ ^]^ and is currently being tested in vivo.^[^ ^173^ ^]^ In 2019, Neuralink^[^ ^174^ ^]^ demonstrated a neural interface with 3072 electrode channels, of which 257 were coated using PEDOT:PSS. Additionally, as described in detail in Section 3, the ability of PEDOT:PSS to blend in with biological substances and create multidimensional, highly biomimetic/biohybrid constructs has enabled seamlessly integrated bioelectronic devices. For example, ultra‐soft hydrogel fabric electrodes^[^ ^175^ ^]^ and biohybrid implants that integrate stem cells have been recently demonstrated,^[^ ^104^ ^]^ opening new horizons for PEDOT:PSS bioelectronics. These findings pave the way for improved, long‐lasting stimulation in medical devices while minimizing harmful byproducts.^[^ ^176^ ^]^ As shown in **Figure** **5**, PEDOT:PSS‐based electrode interfaces have been successfully demonstrated to diagnose/treat a number of conditions linked to the human nervous system, the human cardiac system, and many other systems of the human body. Hence, in the following section, we present an overview of how PEDOT:PSS has been successfully employed in bioelectronic interfaces for both recording and stimulation of human tissues, both in vivo and in vitro.

### 4.1 Interfacing the Human Nervous System

The potential of PEDOT:PSS in bioelectronics has become increasingly evident, particularly for interfacing with the human nervous system — including the brain, the spinal cord, and peripheral nerves. Recording and stimulation of the brain have diverse applications, ranging from preventing epilepsy, monitoring Alzheimer's, and treating Parkinson's or depression.^[^ ^181^ ^]^ In contrast to conventional metal electrodes, PEDOT:PSS offers low impedance (<20 kΩ at 1 kHz), mechanical compatibility, with modulus ranging from 100 to 1000 kPa, and stability in a physiological environment over months—parameters that are essential for high‐fidelity and reliable recording of neural signals.^[^ ^182^ ^]^ In 2003, Cui and Martin et al. created the first neural electrodes with PEDOT:PSS deposited on gold via electropolymerization, leading to a decrease of interface impedance of almost two orders of magnitude compared to uncoated electrodes.^[^ ^183^ ^]^

Ludwig et al.^[^ ^184^ ^]^ showed a non‐human in vivo neural recording with a PEDOT:PSS‐coated silicon‐based device for a period of 6 weeks, demonstrating PEDOT:PSS potential for long‐term recording. In 2011, Khodagholy et al.^[^ ^185^ ^]^ created a Parylene‐C‐based, 4 µm‐thick, highly conformable MEAs with PEDOT:PSS‐coated Au electrodes for highly sensitive, electrocorticography recordings. At first, these conducting polymer coatings suffered from poor electrochemical and mechanical stability, due to overoxidation and poor adhesion to the underlying substrate.^[^ ^186^, ^187^ ^]^ The introduction of strategies such as roughening the substrate material or using adhesion promoters like IrOx enabled long‐term stability of PEDOT:PSS coatings.^[^ ^30^ ^]^ An example is the work of Boehler et al., showing PEDOT:PSS film survival for over 100,000 charging/discharging cycles and 110 days under accelerated aging conditions at 60 °C.^[^ ^188^ ^]^ Similar challenges have been observed with electrodes coated by soft conducting polymer‐based hydrogels, which often exhibit limited operational lifespans due to the tendency to delaminate from the substrate as interfacial water infiltrates, ultimately leading to device failure. To address these adhesion issues, Won et al. also developed a laser‐induced phase separation and adhesion technique of pure PEDOT:PSS hydrogels using a continuous‐wave 532 nm laser.^[^ ^189^ ^]^ The laser was scanned through the transparent substrate and absorbed at the substrate‐PEDOT:PSS interface, inducing partial phase separation and forming micro‐ and nanoscale interlocking structures. The method produced conductive hydrogels with a conductivity of 101.4 S cm^−1^ and spatial resolution down to 5 µm, which could be used to stably record electrophysiological signals in rat brains and hearts for over 3 weeks.

A few years later, Khodagholy et al. further improved the sensitivity of PEDOT:PSS‐based brain interfaces by using an active organic electrochemical transistor (OECT) configuration.^[^ ^190^ ^]^ The device showed a higher SNR by almost 10 dB compared to a standard passive electrode configuration, since small potentials are detected and instantly amplified as an output current signal. The OECTs could reliably measure and distinguish both action potentials and local field potentials, meeting the required bandwidth to measure clinically relevant information in brain recordings. In another work, the researchers showed a highly conformable, nonpenetrating electrode array (Neurogrid) capable of isolating action potentials of individual neurons, showcasing the precision achievable with PEDOT:PSS electrodes.^[^ ^106^ ^]^ Most importantly, the work demonstrated the intraoperative recording of local field potentials in human patients undergoing surgery to treat epilepsy, highlighting the translational potential of these PEDOT:PSS‐based devices, as it is discussed in details in Section 5. Ferro et al. demonstrated a seamless brain machine interface based on individual PEDOT:PSS electrode “roots” (NeuroRoots) with dimensions comparable to those of human axons (i.e., 7 µm wide and 1.5 µm thick) to enable non‐invasive brain recordings.^[^ ^191^ ^]^ The electrodes demonstrated stable chronic recordings in deep‐brain regions for a period of 7 weeks in freely‐moving rats, with no need to adjust their position. Ma et al. developed conformable probes to eliminate the need for skull‐attached rigid support structures and enable recordings from delicate developing tissues, such as those of pediatric patients.^[^ ^192^ ^]^ Stimulation of the brain with flexible, PEDOT:PSS electrodes on polyimide has also been used to improve or restore vision.^[^ ^17^ ^]^ This device performed stable electrical stimulation of the visual cortex of a mice for over a year, with more than ten billion precise electrical pulses.

PEDOT:PSS bioelectronic interfaces have also shown potential in treating disorders of the spinal cord and peripheral nervous system^[^ ^193^ ^]^ through soft and flexible device fabrication that leads to minimally invasive implants with high sensitivity.^[^ ^194^ ^]^ Rochford, Carnicer‐Lombarte et al. created a biohybrid device using PEDOT:PSS that could restore the function of a rat's paw after the nerve was severed.^[^ ^104^ ^]^ They used PEDOT:PSS as an intermediary layer to add living cells on top of the implant before implantation, demonstrating the excellent biocompatibility of PEDOT:PSS compared to other electronic materials. These biohybrid devices are the next step in connecting the body to electronics and offer immense potential for (personalized) healthcare.^[^ ^195^ ^]^ Carnicer‐Lombarte et al. demonstrated the control of individual sensorimotor nerves through a PEDOT:PSS‐based, ultra‐conformable cuff electrode, which found to cause less scarring than clinically approved PDMS silicone cuffs.^[^ ^105^ ^]^ Such PEDOT:PSS‐based ultra‐conformable cuff electrodes enabled precise recording and stimulation of median, ulnar, and radial nerves with minimal surgical disruption. Their low impedance (5.47 ± 4.10 kOhm in saline and 41.64 ± 128.06 kOhm in tissues) and reduced fibrotic response improved chronic stability, offering a durable, less invasive alternative to penetrating nerve interfaces.

Overall, for clinical applications, electrical stimulation of the nervous system requires precise electrical pulses through electrodes that operate reliably, often over long periods, to modulate neural activity. These can be either direct current (DC)—a unidirectional flow of electric charge that can excite/inhibit neural activity—or alternating current (AC)—a pulsed biphasic stimulation commonly within the frequency range of 20–100 Hz.^[^ ^196^, ^197^ ^]^ Increased formation of byproducts (e.g., oxygen radicals) is linked to tissue damage and electrode degradation. This is primarily a concern with DC, particularly when it is continuous and not charge‐balanced, as such conditions drive electrochemical reactions at the electrode–tissue interface. Matter et al. showed that PEDOT:PSS, by increasing the electrode's capacitance, can delay the production of reactive oxygen species (ROS), allowing longer reversibly driven pulse durations during DC stimulation.^[^ ^176^ ^]^

AC, particularly in the form of charge‐balanced biphasic pulses, has been designed to avoid these reactions for chronic use.^[^ ^198^ ^]^ PEDOT:PSS‐based electrodes meet the requirements for precise control of signal parameters, frequency, amplitude, and waveform shape, for safe, long‐term stimulation of the nervous system.^[^ ^199^ ^]^ Despite significant progress, challenges remain in ensuring long‐term stability, mechanical durability, and scalable manufacturing.^[^ ^200^ ^]^ Emerging fabrication techniques and advances in PEDOT:PSS‐based composite materials are expected to play a key role in further advancing clinical translation as well as enable fundamental insights into the function of the brain.

### 4.2 Interfacing the Human Cardiac System

The pacemaker is by far the most successful bioelectronic device that is used in the clinic, and helps millions of individuals around the globe. Electrodes are directly attached on the heart and are controlled by high precision electronics to correct irregular electrical impulses in the myocardium, which are the leading cause of fatal arrhythmia and sudden cardiac death. Traditional electronic pacemakers, while effective, are limited by the lack of physiological responsiveness to changes in heart rate, particularly during exercise.^[^ ^201^ ^]^ The next generation bioelectronic devices for clinical translation in cardiac treatment are designed to provide self‐powered solutions, sensors to respond more naturally to changes in heart rate, and better tissue integration, reducing the risk of tissue inflammation and rejection.^[^ ^202^, ^203^ ^]^ For recording and stimulation, key bioelectronic signals of interest include: normal cardiac activity at 1–1.67 Hz (60–100 heartbeats per minute),^[^ ^204^ ^]^ and pathological cardiac beat 1.67–8 Hz (up to 480 heartbeats per minute for atrial fibrillation).^[^ ^205^ ^]^

The dynamically expanding and contracting environment of the human heart requires a high degree of device stretchability. PEDOT:PSS‐based MEAs, whether in thin film or hydrogel/multidimensional forms, have demonstrated environmental benefits and hold significant promise for future pacemaker technologies. Wang et al. developed a scalable approach for implantable, electrodeposited PEDOT:PSS interface layers connected to an eutectic gallium‐indium alloy confined in an elastomeric matrix as a liquid metal conductor to build interconnects.^[^ ^179^ ^]^ The PEDOT:PSS interface layer reduced the interfacial impedance, enhancing the signal‐to‐noise ratio (SNR) from 69.7 ± 7.4 to 263.2 ± 16.9 mV. Lee et al. showed that a stretchable, active MEAs based on PEDOT:PSS OECTs can further improve ECG signals amplification with SNR at 52 dB.^[^ ^206^ ^]^ Furthermore, in the patent application US20220355103A1, an implantable cardiac pacemaker electrode incorporating a spiral‐shaped metallic wire core and flexible PEDOT:PSS‐coated textile fibers was presented as an effective hybrid design that combines mechanical compliance with stable electrical conductivity. This design is meant to reduce pressure on biological tissues, improving comfort and long‐term wearability in implantable cardiac pacemakers. The use of PEDOT:PSS is central to the invention, as it provides a soft, low‐modulus interface that closely conforms to the heart's surface while maintaining high‐quality signal transmission.

PEDOT:PSS‐based hydrogel and biohybrid electrodes are particularly promising for cardiac disease treatment. Chang et al. developed a PEDOT:PSS hydrogel electrode with conductivity up to 1.6 S cm^−1^, flexibility (Young's modulus of 80 kPa, similar to mammalian heart tissues), and stretchability (270%), leading to high signal‐to‐noise ratio (≈28 dB) electrocardiogram (ECG) recordings and effective pacing with 3.3 V and 5 Hz pulses. The device was further integrated with a wireless power supply module operating through resonant inductive coupling between coils, ensuring device powering without the need for electrical connections.^[^ ^207^ ^]^ However, cardiac dysfunction treatment with pure electrical pacing is, in many cases, not sufficient. Many patients affected by myocardial infarction suffer from infarction‐induced cardiomyocyte loss, resulting in the formation of poorly conductive fibrotic tissues that are electrically decoupled from the viable cardiomyocytes—a disease called atrial fibrillation. In a recent study, Dai et al. demonstrated a shape‐memory PEDOT:PSS‐based hydrogel mesh designed to effectively treat atrial fibrillation.^[^ ^208^ ^]^ The electrically active hydrogel consisted of polyvinyl alcohol and polyacrylamide as the double‐network backbone, and PEDOT:PSS as the conductive component, leading to mechanical stretchability (125%) and conductivity (≈12.5 mS cm^−1^). The mesh injection on the surface of rabbit hearts was found to effectively eliminate atrial fibrillation through bioelectric signal modulation. An even more promising approach for atrial fibrillation and cardiac tissue repair is the use of regenerative bioelectronics that combine stem cells and PEDOT:PSS.^[^ ^209^ ^]^ Roshanbinfar et al. developed an injectable collagen‐PEDOT:PSS hydrogel incorporating human induced pluripotent stem cell (hiPSC) derived cardiomyocytes to promote partial remuscularization of infarcted cardiac tissue.^[^ ^210^ ^]^ The incorporation of PEDOT:PSS enhanced microstructural organization, transforming collagen fibrils into entangled, helical microfibers, resulting in a hydrogel's conductivity of ≈65 mS cm^−1^. When injected into infarcted mouse hearts, the conductive hydrogel reduced post‐myocardial infarction ventricular tachycardia incidence by ∼400 %, restoring arrhythmia levels to those seen in healthy controls. This study demonstrated that collagen–PEDOT:PSS hydrogels not only facilitate electrical pacing but also significantly enhance the maturation and function of transplanted human induced pluripotent stem cell (hiPSC) cardiomyocytes, offering a promising platform for combined electrical intervention and tissue regeneration in arrhythmogenic myocardial injuries.^[^ ^154^ ^]^ These recent results highlight that PEDOT:PSS‐based hydrogels and biohybrid electronics are highly promising for cardiac disease treatment. Next‐generation systems are expected to combine advanced functionalities, such as real‐time sensing, electrical stimulation, and biochemical monitoring into a single platform.

### 4.3 Skin and Wearable Electronics

The concept of electronic skin (e‐skin) has been around since the 1970s, with early examples of flexible and wearable devices integrating silicon (Si) micro‐electro‐mechanical systems (MEMS) on flexible substrates. PEDOT:PSS emerged as a particularly attractive material for applications in contact with skin due to its conductivity and mechanical properties, which more closely resemble those of human skin compared to inorganic alternatives.^[^ ^211^ ^]^ However, one challenge emerged: how to create devices that can conform seamlessly to the skin while maintaining the high conductivity and stability needed for applications? PEDOT:PSS played a crucial role in these developments, opening for the use of conducting polymers in two distinct research directions: stretchable/skin‐like electronics and textile electronics (e‐textile).^[^ ^212^, ^213^, ^214^ ^]^ Here, we focus on PEDOT:PSS devices for skin electronics and we refer the reader to several excellent reviews focusing on textile electronics.^[^ ^215^ ^]^

Initial attempts to develop stretchable organic electronics described the use of PEDOT:PSS films cast on stretchable substrates, such as PDMS. In one of the first reports, Zonyl fluorosurfactant was found to increase PEDOT:PSS wettability of hydrophobic substrates, conductivity, and resistance to tensile strain.^[^ ^214^, ^216^ ^]^ Since 2014, Zonyl products were discontinued due to growing concerns about the harmful effects of long‐chain (per‐ and polyfluoroalkyl substances) PFAS.^[^ ^217^ ^]^ This is an example on how choosing the right additive is crucial for future translation to the market, as highlighted in Section 3.2. Nonetheless, this seminal work on PEDOT:PSS integration on flexible and stretchable substrates opened up the development of conformable sensors and biosensors to monitor health parameters on skin.^[^ ^218^ ^]^ In a more recent example, a study by Olroyd et al. shows the development of a highly stretchable device on PDMS, enabled by the development of a PEDOT:PSS, formulation with 4 wt% polyethylene glycol, 0.05% wt DBSA, and 1 wt% of Capstone FS‐30 and 1 wt% GOPS.^[^ ^107^ ^]^ Many PEDOT:PSS sensors on skin have been developed monitoring biomarkers, such as blood glucose^[^ ^219^ ^]^ and uric acid,^[^ ^220^ ^]^ strain, to monitor motion or breathing,^[^ ^221^ ^]^ and pH, to monitor wound healing.^[^ ^222^ ^]^ Surface functionalization of PEDOT:PSS expanded such sensing capabilities, which are described in detail elsewhere.^[^ ^223^ ^]^

Skin‐contact devices and systems are also used as electrophysiological tools for various applications, such as electromyography (EMG), electrocardiography (ECG), and electroencephalography (EEG). Traditional epidermal electrodes made of silver/silver chloride (Ag/AgCl) have long been considered the clinical standard due to their excellent signal quality and low skin‐electrode impedance.^[^ ^224^ ^]^ However, they rely on ion‐gels that dry out over time, making them unsuitable for long‐term monitoring. Prolonged use can also cause skin irritation, while their rigid structure can lead to discomfort, motion artifacts, and difficulty in integrating into flexible, conformable systems. PEDOT:PSS skin electrodes were first introduced to decrease the impedance of gold electrodes on flexible substrates.^[^ ^225^ ^]^ Since then, many PEDOT:PSS‐based conducting and stretchable electrode constructs have been developed and hold potential for highly accurate, conformable electrode skin electrodes for electrophysiological signal acquisition. Recent developments integrated PEDOT:PSS with different substrates and additives to provide advanced functions, such as imperceptibility, breathability, self‐adhesivity, self‐healing, and even anti‐bacterial properties.^[^ ^226^, ^227^ ^]^ In the quest for unobtrusive and reliable electrodes interfacing with skin, Zucca et al. developed PEDOT:PSS‐based temporary tattoos.^[^ ^228^ ^]^ This concept was expanded to produce devices with ultralow thickness and flexibility while ensuring the patterning resolution, conductivity, and stability needed during device service life.^[^ ^229^ ^]^ PEDOT:PSS hydrogels have also emerged as promising materials for e‐tattoos, combining conductivity, softness, and adhesion to human skin.^[^ ^230^ ^]^

To advance ECG recordings, Gao et al. developed air‐permeable PEDOT:PSS‐based aerogel electrodes conforming well to rough skin and enabling reliable, long‐term, high‐quality ECG signal acquisition during movement (SNR of 22.54 dB versus 19.52 dB for Ag/AgCl gel electrodes, measured on the wrist).^[^ ^231^ ^]^ Alsaafeen et al. showed that PEDOT:PSS‐hydrogel skin electrodes provide sensitive ECG recording, outperforming commercial electrodes (SNR of 44.4 dB versus 26 dB for Ag/AgCl, measured on the wrist).^[^ ^232^ ^]^ Providing electrodes with excellent adhesion and conformability to the skin can substantially decrease the risk of irritation and improve the skin–electrode interface, thus achieving stable and accurate ECG monitoring. Ren et al. developed an adhesive PEDOT:PSS‐based film, leading to excellent conductivity (8.33 S cm^−1^). Integrated into a wearable patch, the adhesive film enabled SNR of 38.85 dB (attached above the heart area) and stable performance in harsh conditions, such as sports and training.^[^ ^233^ ^]^ These results demonstrate the strong potential of PEDOT:PSS‐based soft electronics for future mobile health and wearable electronics applications.

Active devices based on PEDOT:PSS OECTs for ECG recordings have also been demonstrated in 2014 by Campana et al.^[^ ^234^ ^]^ More recently, Yang et al. reported a flexible and integrated OECT array that can perform ECG signal mapping with high spatial and temporal resolution (response time less than 30 µs).^[^ ^235^ ^]^ While these devices require more sophisticated instrumentation to operate, they could provide accurate vital sign monitoring in intensive‐care units for cases where invasive solutions are not recommended, such as for neonatal and pediatric care.

### 4.4 Recording and Stimulating Cells In Vitro

Cell‐based in vitro models are essential tools to advance applications such as drug discovery and safety testing, and tissue engineering. They play a crucial role in advancing basic understanding of disease and in providing human‐relevant alternatives to traditional animal testing, thereby contributing to the 3Rs (replacement, reduction, and refinement) of animal research.^[^ ^236^ ^]^ Conducting polymers, such as PEDOT:PSS, have been introduced to modulate cell parameters, such as adhesion and growth, and to provide in vitro systems with additional functionalities, such as stimulating or recording, as described in detail in a recent review by Pitsalides et al.^[^ ^237^ ^]^

Some of the earliest research questions in the use of conducting polymers to interface with cells were whether they can support cell growth,^[^ ^238^, ^239^ ^]^ followed by whether changes in their redox state could influence cell adhesion and growth in vitro. Experiments using human glioblastoma multiforme cells (T98G) and primary human dermal fibroblasts (hDF) showed that the electrochemical state of PEDOT:PSS has an impact on cell proliferation rate and that such an impact depends on the cell type.^[^ ^240^ ^]^ A follow‐up study focusing on the underlying mechanism showed that PEDOT:PSS behaves as a redox‐controlled ion reservoir, absorbing and releasing ions during reduction and oxidation, respectively. These changes promoted adhesion and proliferation in T98G cells, which are sensitive to small changes in extracellular ion composition and membrane potential, but led to no response in the less excitable hDF cells. Such studies provided important mechanistic insights into how the redox state of PEDOT:PSS modulates the local ionic environment and membrane potential of cells. PEDOT:PSS was subsequently used to provide instructive topographical and electrical cues that support the differentiation and maturation of cells in vitro, with applications spanning neural, cardiac, and osteogenic lineages.^[^ ^241^, ^242^, ^243^, ^244^ ^]^

Evaluating cellular parameters is crucial to developing and assessing in vitro systems, as it enables real‐time monitoring of cell behavior, function, and response to external stimuli. Among the available methods, high‐throughput tools provide rapid and systematic analysis of cellular processes across large sample sets. In particular, microelectrode arrays (MEAs) are widely used to monitor the activity of electrogenic cells in vitro for applications ranging from drug development, toxicity studies, tissue engineering, and developmental biology. Commercial MEAs are typically fabricated using noble metals such as gold. To enhance their performance, the conducting polymer PEDOT:PSS has been introduced as a coating material to reduce electrode impedance (e.g., impedance at 100 Hz from 4305 ± 342 kΩ to 67 ± 2 kΩ for uncoated and coated electrodes, respectively) and improve the signal‐to‐noise ratio (SNR) in recordings of electrogenic cells and tissues.^[^ ^245^, ^246^, ^247^ ^]^ Advances in PEDOT:PSS conductivity and water stability led to the development of PEDOT:PSS‐only MEAs. Adjusting the film thickness led to devices providing high optical transparency, thereby enabling simultaneous high‐sensitivity impedance measurements and optical microscopy.^[^ ^248^ ^]^ Several examples of printed PEDOT:PSS MEAs have also been developed to provide high‐quality signals while abating the cost and complexity of MEAs fabrication.^[^ ^114^, ^249^, ^250^ ^]^

In parallel to MEAs, organic electrochemical transistors (OECTs) based on PEDOT:PSS have emerged as powerful tools to further improve the resolution and SNR of electrophysiological recordings. OECTs offer intrinsic signal amplification, making them particularly well‐suited for real‐time monitoring of cell function and dynamics. Moreover, the bandwidth of PEDOT:PSS transistors is significantly larger than conventional MEAs and can better detect signals at low frequencies.^[^ ^251^, ^252^ ^]^ Response times, on the order of hundreds of microseconds, are adequate to monitor electrophysiological signals with SNRs reaching 12–25 dB in cell culture recordings.^[^ ^253^ ^]^ OECT arrays, in addition to temporal resolution, also provide spatial mapping capabilities, enabling to study of individual cell responses and intercellular interactions through the detection of propagating bioelectronic signals. Another successful example of PEDOT:PSS‐based OECTs applied to analysis of in vitro systems is the evaluation of the integrity of barrier tissue,^[^ ^254^ ^]^ as first demonstrated by Jimison et al. in 2012.^[^ ^255^ ^]^ This capability holds significant promise for applications in drug permeability assays, disease modelling, and the development of physiologically relevant organ‐on‐chip platforms.

Beyond PEDOT:PSS films, 3D in vitro bioelectronic systems based on PEDOT:PSS have been reported as highly biomimetic interfaces that eliminate the mismatch between 3D/static electronics and 3D/dynamic biology.^[^ ^256^, ^257^ ^]^ Savva et al. demonstrated PEDOT:PSS‐based scaffolds that can be fine‐tuned to host human stem cell cultures and monitor their growth with impedance spectroscopy.^[^ ^123^ ^]^ Conducting hydrogels made of PEDOT:PSS are also emerging as highly biomimetic, 3D in vitro systems. Borah et al. used an electrically conductive silk/PEDOT:PSS‐based hydrogel to encapsulate induced pluripotent stem cells and differentiate them into a 3D cortical neuron network.^[^ ^142^ ^]^ These developments show the potential of PEDOT:PSS to enable truly biomimetic, 3D bioelectronic systems capable of monitoring and stimulating advanced biological processes in vitro, thereby significantly contributing to the development of novel therapeutic strategies for a range of diseases.

To summarize the key considerations discussed in this perspective, **Table** **1** presents the most critical parameters for designing organic electronic components and devices, emphasizing PEDOT:PSS's versatility across various categories of bioelectronic interfaces.

**Table 1:** Summary of key properties to consider for bioelectronic interfaces based on PEDOT:PSS components.

Property	Value range	Remarks	Application	Refs.
**Thin Films**—*solution processing and micropatterning*				
Electrical conductivity	100–15 000 [S cm^−1^]	Dependent on processing additives and post‐deposition treatments.	MEAs and OECTs; Recordings and stimulation of human cells, tissues, and organs. Connection with back‐end electronics.	[^11^, ^12^, ^13^]
Ionic conductivity	10^−6^–10 ^−3^ [cm^2^ V^− 1^ s^− 1^]	Dependent on specific ions. Similar to the ionic mobility of ions in bulk water. Strongly depended on additives and crosslinkers.	Direct interface with cells. Durable under long‐term pulsing.	[13, 71, 258]
Capacitance (volumetric)	39 [F cm^−3^]	Volumetric interaction of ions and electrons. Films are swollen with hydrated ions reversibly.	Low impedance MEAs and OECTs, suitable for high SNR electrodes for recordings and high CIC electrodes for stimulation.	[140]
Transparency	80%–95% (thickness 200–30 nm)	Visible spectrum transparency (λ = 450–750 nm).	In vitro cell culture for simultaneous electrophysiology and high‐resolution optical microscopy.	[247, 259]
Adhesion on substrates	Strong–durable	Selection of processing additives for adhesion on a range of flexible and stretchable substrates.	Conformable, durable implants and interfaces with human tissues; MEAs in vitro cultures; Skin electronics.	[20, 75]
Stretchability	>50%	Flexibility in design; Retain function under strain.	Skin electronics; Cardiac Implants.	[107]
**3D Constructs and Electrically Active Hydrogels**—*biohybrid, biomimetic, additive manufacturing*				
Electrical Conductivity	10^−5^–10^2^ [S cm^−1^]	Depended on the method of preparation (Section 3.3).	3D MEAs; In vitro systems; Recordings and stimulation of human tissues (e.g., for repair and functional restoration).	[143, 145, 260]
Ionic Conductivity	10^−3^–10 ^−2^ [S cm^−1^]	Depended on electrolyte composition and cell/tissue environment.	Standardization of electrochemical characteristics for 3D hydrogels.	[145]
Capacitance	10^−7^–10^2^ [F]	Depended on the method of preparation (section 3.3). Also depended on thickness and porosity and conducting loading for electrically active hydrogels.	Standardization of electrochemical characteristics for 3D hydrogels.	[145]
Transparency	>10%–50%	Depended on the method of preparation (section 3.3). Also depended on thickness, porosity and conducting loading for electrically active hydrogels.	In vitro 3D cell culture imaging with high resolution, fluorescence microscopy.	[260]
Adhesion on substrates	< 7 kPa for PEDOT:PSS composites	Highly hydrated constructs, with ultrasoft properties not compatible with rigid substrates. Chemical bonding on soft substrates.	Prevent delamination in contact with biological fluids and ensure connection to back‐end electronics for implanted systems.	[143, 260]
Viscoelasticity	G’ = 1–100 [kPa]	Tunable storage (elastic) modulus and viscous modulus depended on the method of preparation and processing.	Biohybrid implants and electronics. Cell encapsulation and stem cell/ regeneration studies.	[140, 261]

## 5 Clinical Trials and Translational Challenges and Opportunities

Recording neural activity during neurosurgical interventions is an invaluable tool for both improving patient outcomes and advancing our understanding of neural mechanisms and organization. The electrochemical, mechanical, and processing properties of PEDOT:PSS electrodes, as discussed in the previous sections, have proven beneficial in clinical applications. In **Table** **2**, we summarize the past and current clinical trials that have produced promising results and paved the way for further clinical translation of PEDOT:PSS‐based bioelectronic devices. So far, five clinical applications with a total of more than 50 human subjects have been recorded with promising results as described in the following paragraphs. It is worth noting here that clinical applications in humans can be distinguished as registered clinical trials and trials that are approved from Institutional Review Boards (IRBs) for acute intraoperative human subject recordings during operations (e.g., the UC San Diego Health Institutional Review Board—IRB).

**Table 2:** Human studies and clinical trials using PEDOT:PSS (2015–2025).

Year	Type	Device	Human subjects	Human context	Notes	Refs.
2015	IRB‑approved human study^*^	NeuroGrid (high density PEDOT:PSS electrodes)	NR	Epilepsy surgery; intra‑operative acute recordings	Demonstrated high‑resolution ECoG and unit activity from human cortex	[106]
2016	IRB‑approved human study	NeuroGrid (high density PEDOT:PSS electrodes)	*N* = 5	Intra‑operative recordings in awake and anesthetized patients	Recorded LFPs and single‑unit—like activity; includes intra‑operative photos and IRB acknowledgments	[262]
2018	IRB‑approved human study	PEDOT:PSS µECoG micro‑ and macro‑dot arrays	*N* = 4	Epilepsy/tumor surgery; intra‑operative acute recordings (awake & anesthetized)	Benchmarked PEDOT:PSS vs clinical Pt/PtIr; stimulus‑locked activity in humans	[263]
2020	IRB‐approved human study	NeuroGrid (high density PEDOT:PSS electrodes)	*N* = 4	Epilepsy surgery; intra‑operative acute recordings	Bonded conformable neural probes to rigid amplifiers without metal‐metal soldering.	[264]
2021	IRB‑approved human study	High‑density PEDOT:PSS micro‑ECoG arrays	*N*≈36	Intra‑operative cortical recordings across multiple hospitals	Identified three classes of microscale events; rates modulated by stimuli/meds; includes PEDOT:PSS setup figure	[265]
2025	Registered clinical trial^**^	Panaxium micro‑ECoG using PEDOT:PSS	NR	Awake glioma surgery; intra‑operative acute use	Safety and signal quality endpoints; compares PEDOT:PSS micro‑ECoG vs standard macro‑ECoG	NCT06408428

* IRB‐approved studies: short‐term intraoperative recordings under ethics approval, not registered as interventional clinical trials.

** Clinical trial (NCT06408428): registered on ClinicalTrials.gov; ongoing as of 2025.

The first clinical application was performed in 2015 and used a high‐density array of PEDOT:PSS‐based, ultra‐conformable, electrodes and OECTs (i.e., the NeuroGrid, discussed in detail in chapter 4.1).^[^ ^106^ ^]^ The NeuroGrid device was also used in 2016 for electrocorticography (ECoG) recordings from subjects undergoing epilepsy surgery.^[^ ^262^ ^]^ The study acquired high‐quality electrophysiological signals in anesthetized and awake human subjects (five subjects in total—three female and two male). In 2018, a clinical application was demonstrated with PEDOT:PSS microelectrode arrays for intraoperative monitoring of the human brain.^[^ ^263^ ^]^ PEDOT:PSS microelectrodes were used to perform intraoperative recordings in both anesthetized patients and patients undergoing clinical mapping of eloquent cortex during epilepsy and tumor resection surgery (four individuals). The study showed that PEDOT:PSS electrodes record comparable activity to current clinical electrodes—i.e., macrodot Pt clinical electrodes versus microdot PEDOT:PSS electrodes—during two different states when the electrodes were implanted on the anterior superior temporal gyrus. In 2020, a high‐density NeuroGrid array that used a mixed‐conducting particulate composite (MCP) as the electrical and mechanical interface was used for acute ECoG recordings.^[^ ^264^ ^]^ The MCP was made of PEDOT:PSS microparticles dispersed in a chitosan–sorbitol ion‐conducting polymer matrix, forming a soft, biocompatible, anisotropic conductor that bonded conformable neural probes to rigid amplifiers without metal‐metal soldering. For intraoperative human recordings, MCP‐bonded NeuroGrids were inserted through the small burr hole created for deep‐brain‐stimulation electrode placement and laid directly on the exposed cortical surface. These arrays safely recorded high‐signal‐to‐noise local field potentials and gamma oscillations from the human cortex for up to 30 min during surgery, demonstrating that the PEDOT:PSS‐based MCP interface can provide stable, high‐resolution neural recordings from human subjects. In 2021, a clinical trial used high‐density PEDOT:PSS microelectrodes in four different centers to record across 37 patients undergoing craniotomies for surgical resection of tumor and epilepsy (mean =  39.7 years old, ranging from 22 to 62; 18 female and 19 male).^[^ ^265^ ^]^

In invasive neural recordings, two main signal types are analyzed: action potentials (spikes) and local field potentials (LFPs). Spikes are brief electrical events generated by individual neurons, occurring at firing rates of tens of hertz but containing high‐frequency components between 0.1–7 kHz, thus requiring high‐pass filtering in this range. In contrast, LFPs reflect the summed transmembrane currents of neuronal populations, spanning ∼0.1–200 Hz and commonly divided into frequency bands such as delta, theta, alpha, and gamma. These frequency distinctions are essential for accurate separation and analysis of spikes and LFPs, as discussed by Thakor et al.^[^ ^266^ ^]^ Importantly, PEDOT:PSS microelectrode arrays (MEAs) have demonstrated the capability to record both LFP dynamics (typically <200 Hz) and single‐unit activity (1–10 kHz) with high sensitivity.^[^ ^266^ ^]^ The high sensitivity of PEDOT:PSS electrodes (30 µm in diameter) revealed microscale neural signals distinct from the high and low frequency signals that are usually the only ones that are shown with conventional electrode recordings. An ongoing clinical trial in France aims to assess the safety and the quality of the signals provided by newly developed micro ECoG electrodes provided by the company Panaxium, which are based on PEDOT:PSS (ref—trials.braintumor.org/trials NCT06408428). The trial targets patients suffering of gliomas during resection surgery performed in an awake condition. The main questions that the trial aims to answer are: what is the quality of PEDOT:PSS microECoGs recordings compared with recordings with traditional macroelectrodes, the practicality of microelectrodes use as perceived by neurosurgeons, and the ability to record multi‐unit activity and tumor infiltration, amongst others.

These clinical trials revealed some challenges that need to be addressed for the wider clinical translation of these technologies to the clinic, such as standardized sterilization approaches, device stability, and approvals. The effective sterilization that renders the devices free from pathogenic agents is one important aspect to transition implantable PEDOT:PSS technologies to the clinic. For example the first clinical application of PEDOT:PSS in 2015^[^ ^106^ ^]^ (see Table 2) relied on ethylene oxide sterilization, a method that is used for sterilization of disposable plasticware in bulk, and not directly available in most hospitals. This was the case also for the clinical application of the NeuroGrids in 2016, where NeuroGrids, headstages, and associated cables were sterilized using a 97 to 100% ethylene oxide at 50–60 °C for 4 h followed by 12 h of detoxification. Uguz et al. proved that sterilization of PEDOT:PSS electrophysiology devices (i.e., both MEAs and OECTs) can be performed using an autoclave.^[^ ^267^ ^]^ These results were verified simultaneously in two independent laboratories and found that autoclaving is a viable sterilization method, leaving morphology unaltered and causing only minor changes in electrical properties. While several methods exist for the sterilization of implantable medical devices, autoclaving is the most frequently used, by exposing them to high‐pressure steam typically at a temperature of 121 °C for several minutes. The simplicity of the procedure and the low cost of the equipment make this method widely available among clinics worldwide. In fact, the clinical application in 2018 used autoclave sterilization for implantable PEDOT:PSS electrodes.^[^ ^263^ ^]^ 3D atomic force microscopy topography showed that the PEDOT:PSS film after autoclave sterilization has a smooth and uniform morphology with the absence of voids and stable electrical performance, similar to the one before autoclaving.

Device fabrication should also be tailored for clinical translation. In the clinical application study of 2021 the authors describe PEDOT:PSS electrode manufacturing, followed by acute electrode placement procedures performed in the operating room (OR).^[^ ^265^ ^]^ First, they observed that using thicker (14 µm) parylene C layers for subdural recording in humans resulted in additional mechanical stability that improved device yield throughout post‐fabrication evaluation, packaging, shipping, sterilization, and use in the OR. Second, they used graded widths of parylene C from the connector region to the microcontact region to distribute stresses more evenly and avoid 90° cuts or patterns in parylene C, which can nucleate cracks and tears under stress. Third, they found that incorporating perfusion holes through the grid can improve conformal contact between the grid and the brain surface, which in turn resulted in higher fidelity recordings. At last, they noted that functional channels on the brain surface had higher yield when the operating neurosurgeon had more experience using the device. These findings represent a significant step forward, paving the way for the wider clinical implementation of PEDOT:PSS devices.

## 6 Outlook and Conclusion

We presented an overview of PEDOT:PSS as a key material in bioelectronics, tracing its development from early polymer research to the emergence of conducting polymers, and the development of PEDOT:PSS in the very late 1980s. We reviewed its evolution from the 1990s, and onwards, emphasizing the milestone research directions that led to the current form of PEDOT:PSS used in bioelectronics. PEDOT:PSS offers distinct advantages over traditional electronic materials due to its mixed ionic‐electronic conductivity, aqueous processability, and tunable properties through additives and post‐processing. We detailed how its performance can be optimized via chemical modification and fabrication approaches, enabling the production of multifunctional and multidimensional constructs – from particles to thin films and 3D bio‐integrated structures. We reviewed the current landscape of PEDOT:PSS‐based bioelectronic interfaces that show strong potential to enter the clinic in the coming years, including neural, cardiac, and epidermal devices. Ongoing research in biohybrid electronics, encapsulation strategies, and soft interconnects is progressively addressing these barriers as well as expanding the capabilities of in vitro systems that mimic the human body environment with high fidelity. As these technical challenges are resolved, PEDOT:PSS is positioned as a strong candidate for clinically viable, stable, and biocompatible bioelectronic systems. The current and past clinical trials that we review here, show great promise and pointing to specific challenges and opportunities for wider clinical translation. Regulatory validation remains a key barrier, requiring formulation and device‐specific assessment of safety, performance, and biocompatibility. Continued multidisciplinary efforts in materials engineering, electronic device design, system integration, and clinical validation will be critical for translating PEDOT:PSS technologies from research labs to the clinic. Overcoming these challenges paves the way for the widespread clinical adoption of PEDOT:PSS‐based bioelectronic systems, which have the potential to enhance quality of life and broaden access to advanced healthcare.

To conclude, this perspective aims to provide a foundation for both newcomers to bioelectronics and researchers already working within the field across different disciplines. As the field continues to evolve rapidly, integrating diverse concepts to identify new opportunities can be challenging. Through this overview, we hope to foster stronger interdisciplinary collaborations that will further advance organic bioelectronic technologies for improved diagnostics, therapeutics, and overall human health.

## Conflict of Interest

The authors declare no conflict of interest.
